# Agreement study between color and IR retinal images based on retinal vasculature morphological parameters

**DOI:** 10.1186/s12886-018-0997-6

**Published:** 2019-01-21

**Authors:** Aqsa Ajaz, Behzad Aliahmad, Himeesh Kumar, Marc Sarossy, Dinesh K. Kumar

**Affiliations:** 1 School of Engineering, RMIT University, Melbourne, Australia; 2 Department of Ophthalmology, Alfred Healthcare, Melbourne, Australia

**Keywords:** Infrared scanning laser ophthalmoscope, Optical coherence tomography, Retinal image vasculature assessment software, Integrative vessel analysis

## Abstract

**Background:**

Color fundus photography have been extensively used to explore the link between retinal morphology changes associated with various disease i.e. Diabetic Retinopathy, Glaucoma. The development of multimodal imaging system that integrates Infrared Scanning Laser Ophthalmoscope (IR-SLO) and Optical Coherence Tomography (OCT) could help in studying these diseases at an early stage. The aim of this study was to test the agreement between the retinal vasculature parameters from the Infrared images obtained from optical coherence tomography and color fundus imaging.

**Methods:**

The IR and Color retinal images were obtained from 16 volunteer participants and seven retinal vessel parameters, i.e. Fractal Dimension (FD), Average Angle (ABA), Total Angle Count (TAC), Tortuosity (ST), Vessel/Background ratio (VBR), Central Retinal Arteriolar Equivalent (CRAE) and Central Retinal Venular Equivalent (CRVE) were extracted from these retinal images using Retinal Image Vasculature Assessment software (RIVAS) and Integrative Vessel Analysis (IVAN).

**Results:**

The Bland Altman plot was used to investigate the agreement between the two modalities. The paired sample t-test was used to assess the presence of fixed bias and the slope of Least Square Regression (LSR) line for the presence of proportional bias.

The paired sample t-test showed that there was no statistically significant difference between Color and IR based on retinal vessel features (all *p* values > 0.05). LSR also revealed no statistically significant difference in the retinal vessel features between Color and IR.

**Conclusion:**

This study has revealed that there is a fair agreement between Color and IR images based on retinal vessel features. This research has shown that it is possible to use IR images of the retina to measure the retinal vasculature parameters which has the advantage of being flash-less, can be used even if there is opacity due to cataract, and can be performed along with OCT on the same device.

## Background

Retinal imaging is used for diagnosis and monitoring of number of ocular and systemic diseases i.e. Diabetic Retinopathy, Glaucoma and risk of stroke event [1]. There are multiple modalities for retinal imaging and the three routinely used are: eye fundus photography, fluorescein angiography and optical coherence tomography (OCT) while some of the recent advancements in the field include hyperspectral imaging and infrared imaging [2].

Recent technological advances have seen enhanced capabilities and significant reduction in the price of OCT leading to their widespread use in busy ophthalmology practices. OCT captures high resolution 3D cross-sectional image of retina, making it suitable for detecting a wide spectrum of retinal disease such as wet age-related macular degeneration and Macular Edema. Introduction of advanced imaging systems that integrates OCT and Infrared Scanning Laser Ophthalmoscope (IR-SLO) into a single device offers various advantages over other imaging systems. The device provides pixel to pixel correspondence between OCT and IR-SLO and improves the quality of illumination by removing the out of focus scattered components of the reflected light [3, 4] enabling the clinicians to have view of both the morphological changes and structural alterations in the retina**.** Other advantage is that infrared (IR) imaging does not require a flash and is less traumatizing to the patient. Another potential advantage is higher media opacity penetration compared than white light making it suitable for imaging patients with cataracts. It also has the potential to provide deeper visualization of the retina, including the choroidal vessels as it comprises longer wavelengths compared to the Green channel which is commonly used in color fundus photography [3, 5].

Eye fundus images have been extensively studied to determine the association of disease with changes in retinal vascular. Number of parameters and geometrical characteristics have been investigated and used as biomarkers to quantify the changes in the retinal vasculature; fractal dimension, tortuosity, vessel caliber and branching angle [6]. Fractal analysis based association has been determined for aging [7], diabetic retinopathy, detection of open angle glaucoma [8] as well as cerebral infarction [9]. Retinal vascular tortuosity has been identified as a biomarker for early stages of microvascular damage in diabetic patients [10], hypertensive retinopathy, stroke and retinopathy of prematurity [1]. Vessel caliber changes have association with early detection of cardiovascular diseases and hypertension [11]. Variations in retinal vasculature caliber has also been reported to be associated with stroke, especially cerebral infarction [12]**,** diabetic retinopathy [13, 14], age related macular degeneration [15–17], obesity [18] and retinal vein occlusion [19]. All the retinal vasculature studies have been performed using color fundus images. While color fundus imaging is now routine, the image quality is poor for people with cataract [20] and is unsuitable for people hypersensitive to flash of light [3].

IR image is obtained during OCT examination and is used to view the structure of retina, sub-retinal lesions, accumulation of fluid in retina of patients with choroidal neovascularization [21], age related macular degeneration [22], stargardts disease [23] and provide information about the site of leakage and leakage patterns. These images can detect pathologies even in presence of haemorrhages and cataract that may be undetected on other imaging system [24–26] and thus imaging using IR can overcome some of the limitations in the color fundus imaging. However, these images have never been explored to replace the color fundus images and have not been considered for investigating the changes in the retinal vasculature morphology with disease.

The aim of this study was to validate the use of IR images obtained during routine OCT for investigating the retinal vasculature morphology. We have tested the hypothesis: ‘The morphological features of vasculature in the color fundus images and IR images of the retina are equivalent’. The IR and color eye-fundus images of volunteers were recorded and the baseline retinal vasculature features from both the images were extracted and tested for the agreement. Statistical analysis was also performed to investigate the difference between the two images.

## Materials

Retinal photographs of healthy volunteers were collected from North West Eye Specialists, Gladstone Park, Melbourne, Australia. The experimental protocol was approved by RMIT University human experiments ethics sub-committee and performed in accordance with Helsinki accord 1986 (modified 2004). The experimental protocol was described in plain language to each participant and written consent was obtained prior to the experiment.

Optic disc centered Color and IR retinal images were obtained from both eyes of each participant without pupil dilation. Color images were taken using Canon CR-1 (Canon, Tokyo, Japan) and IR images were obtained by Spectralis SD-OCT (Heidelberg Engineering, Heidelberg, Germany) with an integrated IR-SLO imaging system. Sixteen unpaid healthy volunteers participated in this study. The mean age of the volunteers was 37.66 (±11.28) years with range 24 to 52 years. All volunteers self-declared themselves as healthy, non-smokers, moderately active and with no history of diabetes, hypertension or retinopathy. None of the participants had clinically reported opacity due to cataract or other causes. A total of 60 images (30 Color and 30 IR) were collected, but images obtained from 3 volunteers were discarded due to motion artefacts leaving 48 images which were used for analysis.

## Methodology

**Fig. 1:**
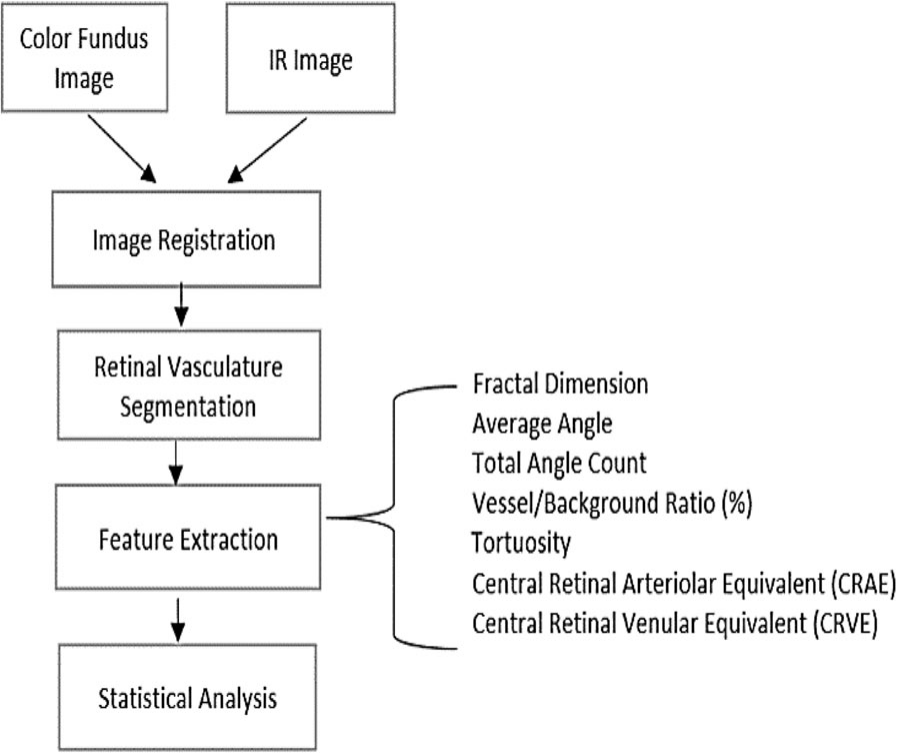
Block diagram of the proposed method for comparison between Color and IR image

Figure 1 shows the block diagram of the proposed method for comparison of Color fundus and IR images. The method involves three main processing steps. 1) Image Registration (alignment) 2) Image Segmentation and 3) Extraction of Retinal Vasculature Parameters.

## Image registration

**Fig. 2:**
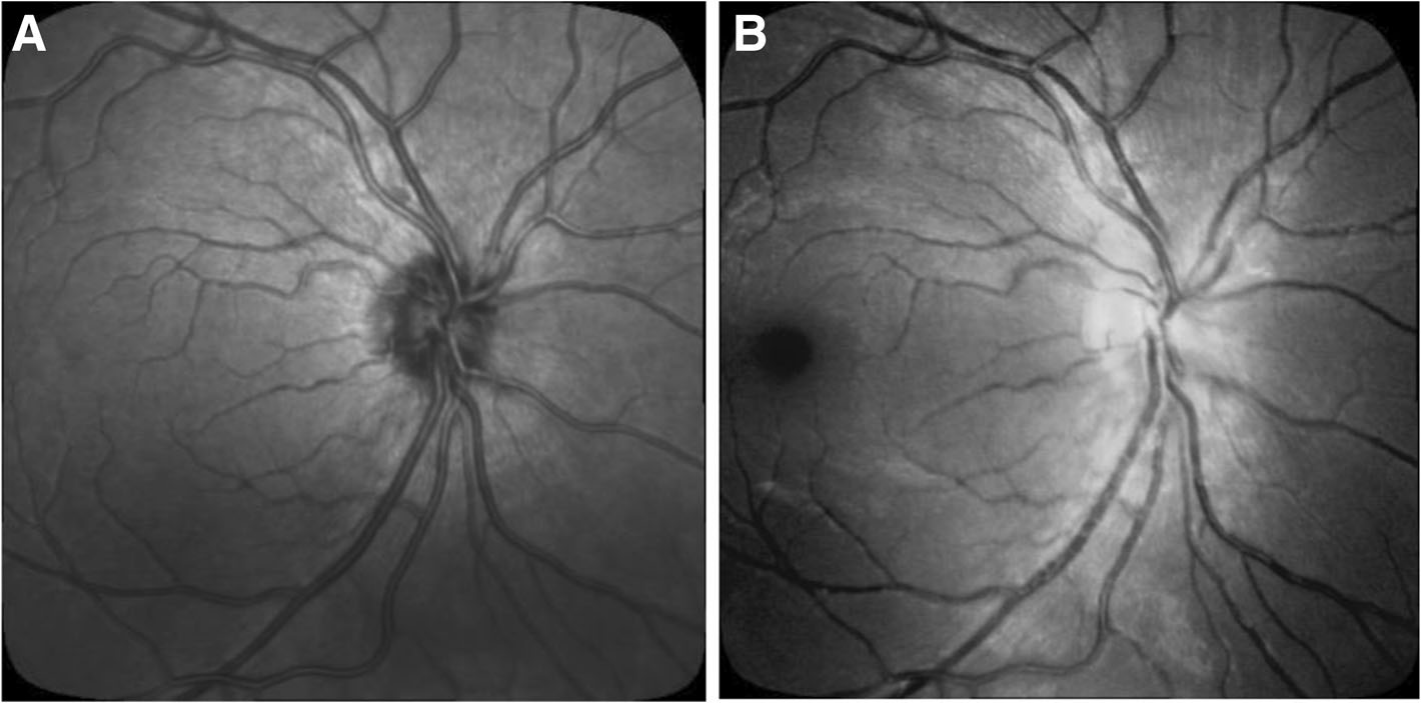
Images after affine registration: (**a**) Color Fundus Image; (**b**) IR Image

Registration (alignment) is a preliminary step when analysing images captured from two different imaging modalities with different fields of view. IR images obtained from Spectralis SD-OCT have a wider field of view of retina compared to fundus photography and for effective comparison between the two images; these were registered and cropped to ensure that the two images share identical region of interest and have a maximum overlap. Image registration was performed using the i2k Retina software, Dual Align, v.2.3.1. To prevent geometric distortion and alterations to the morphology of retinal vessels, only linear affine transformations were applied to the images. After proper alignment, the images were cropped using a bounding rectangle to focus the analysis to the region of interest. Figure 2 shows an example of two registered images. For color image (Fig. 2.a), the green channel was selected as it is recognised to provide the maximum contrast between the vessel and background.

## Image segmentation

**Fig. 3:**
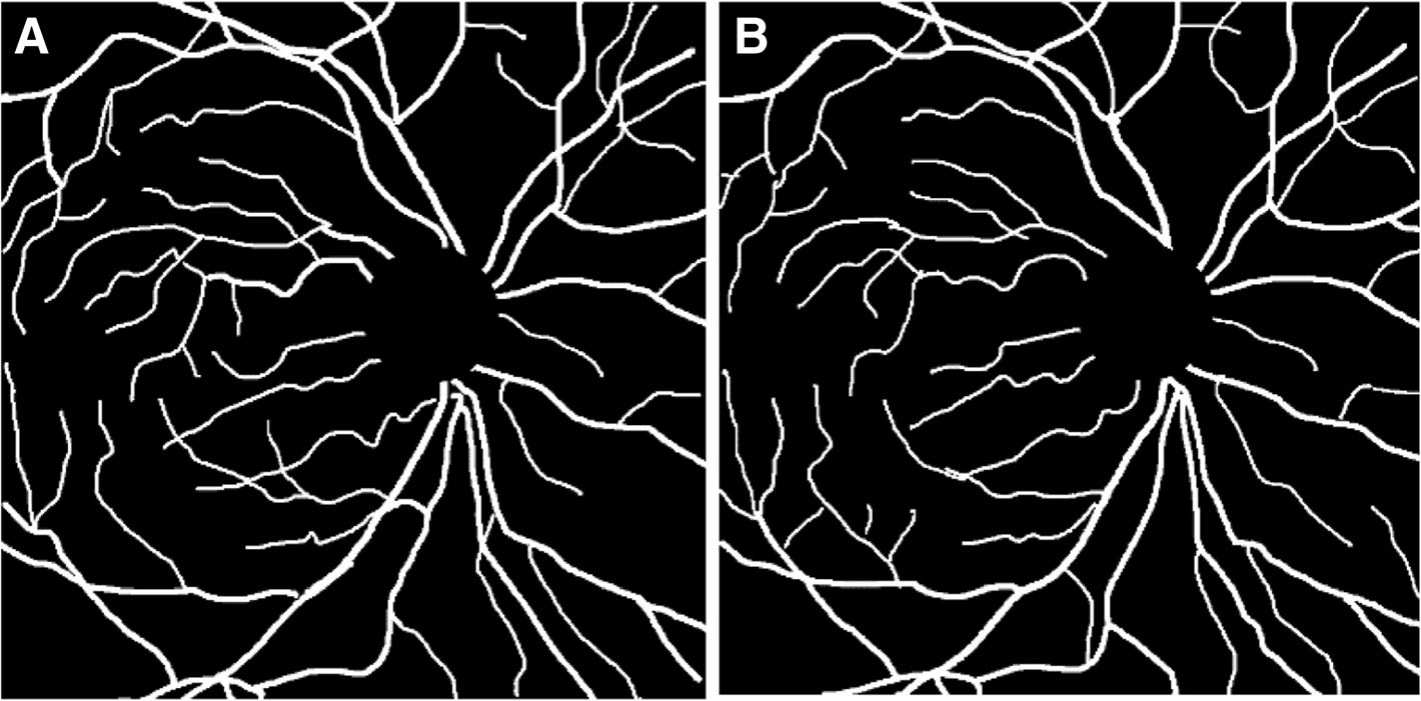
One set of Manually segmented Images: (**a**) Color Fundus Image; (**b**) IR Image

Extraction of retinal vessels was the first step in evaluating the retinal vasculature parameters. As, IR images suffer from low contrast and blur this could have led to segmentation errors. In order to avoid this manually segmented color and IR images of retina were generated. Two independent experienced graders using Wacom, Intuos touch pen tablet manually segmented the images to establish the ground truth. Figure 3 shows example of manually segmented color and IR images. The correlation coefficient between the two set of ground truth images was *r* = 0.85.

## Extraction of retinal vasculature parameters

Retinal vessel parameters were calculated from the segmented images using functions from the unsupervised Retinal Image Vasculature Assessment Software (RIVAS v1.1, Australia) which was previously developed for color fundus images [27]. The five retinal vessel parameters: Fractal dimension (FD), Simple Tortuosity (ST), Average Branching Angle (ABA), Total Angle Count and (TAC) and Vessel to Background Ratio (VBR) (%) were evaluated using the segmented images. In this study, FD which is an important parameter that indicates the vascular network complexity was calculated on the skeletonized image using the Binary Box Counting method. ABA was defined as the average of the acute angle between two daughter vessels and ST was measured as the ratio of the actual length of the vessel to the shortest (Euclidean) distance between the two endpoints within the same segment.

Two retinal vessel diameter summary measurements, i.e. Central Retinal Arteriolar Equivalent (CRAE) and Central Retinal Venular Equivalent (CRVE), were measured using IVAN (University of Wisconsin, Madison, WI, USA) using the revised Knudtson-Parr-Hubbard formula. The diameter of six largest arterioles and venules were located in zone corresponding to 0.5–1 disc diameter from optic disc and these were used to calculate CRAE and CRVE [28].

## Statistical analysis

This research has tested the agreement between the baseline retinal vasculature parameters obtained using color and IR optic disc centred retinal images of healthy volunteers. Seven retinal vasculature parameters used for comparing the two modalities were: FD, ABA, TAC, ST, VBR, CRAE and CRVE. The statistical analysis was performed in MedCalc 10.0.2.0 (MedCalc Software Ostend, Belgium).

Bland Altman’s plot (BA) plots were used to investigate the degree of agreement between the retinal features extracted from Color and IR images obtained from two different imaging systems. Therefore, the difference between retina vascular parameters obtained from IR images and the corresponding parameters from color images as ground truth was plotted against average of the two parameters. This plot also provides information about the presence of systematic biases and outliers. The mean and the standard deviation (SD) were used to define a 95% limits of agreement (LOA) i.e. LOA = mean ± 1.96SD. This shows the error bars representing the 95% confidence interval (CI) for upper and lower LOA [29, 30] for each parameter.

Paired sample t-test was performed to determine whether the mean difference between the two corresponding parameters (Colour and IR) is zero and investigate the presence of fixed bias. If the *p* value from the *t* -test is greater than the level of significance (i.e. α = 0.05), this indicates that there is no fixed bias and hence there is no evidence that lack of disagreement between the two methods.

BA scatter plot along with the 95% CI was constructed and the trend line was obtained to determine the presence of proportional bias which is for the case when there is an imbalance of the data points in any one side of the line. The slope of least square regression line was obtained. A non-statistically significant *p*-value from the regression line indicated the absence of proportional bias. A presence of proportional bias indicated that the discrepancies between the two measurements were not uniform throughout the range of measurements and therefore no agreement between the two measurements.

In addition, Cohen’s kappa coefficient (κ*)* was calculated to measures the strength of agreement between the vessel features obtained from color and IR images. Kappa Coefficient measures the agreement between two categorical variables X and Y and result can be interpreted as follows: values ≤0.20 as indicating poor agreement and 0.20–0.40 as fair agreement, 0.40 to 0.60 as moderate, 0.60 to 0.80 as good and 0.80–1.00 as perfect agreement [31].

## Results

In order to verify that the differences were normally distributed, the Kolmogorov-Smirnov test was performed before the BA plot.

Table 1 shows the mean, 95% CI (BA plot), the *p* values for *t*-test, *p* values for Linear Regression and the Cohen’s kappa (κ) based on the comparison between the five features obtained by RIVAS from color fundus and IR images that were manually segmented and referred to as the ground truth.

**Table 1:** Agreement analysis between the two sets of segmented Color and IR images based on obtained retinal vascular parameters

	Parameter	Mean Difference	LOA	*p* value *	*p* value**	κ ***
Ground Truth Set 1	FD	−0.003	0.053 to − 0.058	0.63	0.88	0.59
	VBR (%)	0.001	0.030 to −0.029	0.79	0.90	0.42
	ABA	1.2	13.3 to −10.9	0.34	0.73	0.28
	TAC	− 5.1	17.7 to −27.9	0.05	0.14	0.05
	ST	−0.005	0.023 to −0.033	0.10	0.80	0.20
Ground Truth Set 2	FD	0.00	0.060 to −0.059	0.96	0.28	0.25
	VBR (%)	0.00	0.027 to −0.027	0.97	0.64	0.29
	ABA	1.50	15.0 to −12.1	0.31	0.11	0.22
	TAC	0.90	22.0 to −20.3	0.68	0.78	0.04
	ST	0.003	0.079 to −0.073	0.69	0.13	0.25

*p-value of one sample t-tests (comparing between mean difference and zero value) to indicate presence of systemic bias

**p-value of Regression Analysis to indicate presence of proportional bias

***κ Cohen’s kappa value to indicate the level of agreement color and IR images

The mean difference between the Color and IR for the first set of ground truth based on RIVAS vessel features were: FD = − 0.003 (95% LOA 0.053 to − 0.058), VBR = 0.001 (95% LOA 0.030 to − 0.029), ABA = 1.2 (95% LOA 13.3 to − 10.9), TAC = − 5.1 (95% LOA 17.7 to − 27.9) and ST = − 0.005 (95% LOA 0.023 to − 0.033).

When comparing the RIVAS vessel features for the second ground truth images, we observed that the mean difference between Color and IR for features were: FD = 0.000 (95% LOA 0.060 to − 0.059), VBR = 0.000 (95% LOA 0.027 to − 0.027), ABA = 1.50 (95% LOA 15.0 to − 12.1), TAC = 0.90 (95% LOA 22.0 to − 20.3) and ST = 0.003 (95% LOA 0.079 to − 0.073).

The paired sample *t*-test showed that there was no statistically significant difference between Color and IR based on retinal vessel features (all *p* values > 0.05) indicating the absence of fixed bias.

The slope of the least square regression line was used to test for the presence of any proportional bias. For the first set of ground truth, *r,* the linear regression values, for RIVAS features were all correlated negatively: FD = − 0.021 (95% LOA -0.308 to 0.266); VBR = − 0.028 (95% LOA -0.514 to 0.457); ABA = − 0.094 (95% LOA -0.672 to 0.4829); TAC = − 0.533 (95% LOA -1.262 to 0.194) and ST = − 0.072 (95% LOA -0.686 to 0.542). For the second set of ground truth, these were correlated negatively with linear regression values, *r*: FD = − 0.31 (95% LOA -0.900 to 0.280); VB = − 0.113 (95% LOA -0.611 to 0.384); ABA = − 0.489 (95% LOA -1.110 to 0.132). However, TAC = 0.096 (95% LOA -0.618 to 0.811) and ST = 0.568 (95% LOA -0.201 to 1.338) showed positive correlation. The absence of proportional bias was observed in terms of all vessel features of both the set of images.

The *p* values of both, the *t*-test and linear regression, shows the absence of fixed or proportional bias and confirms that there is no statistically significant difference in these five-retinal vessel features of the colour and the IR images of the retina.

**Fig. 4:**
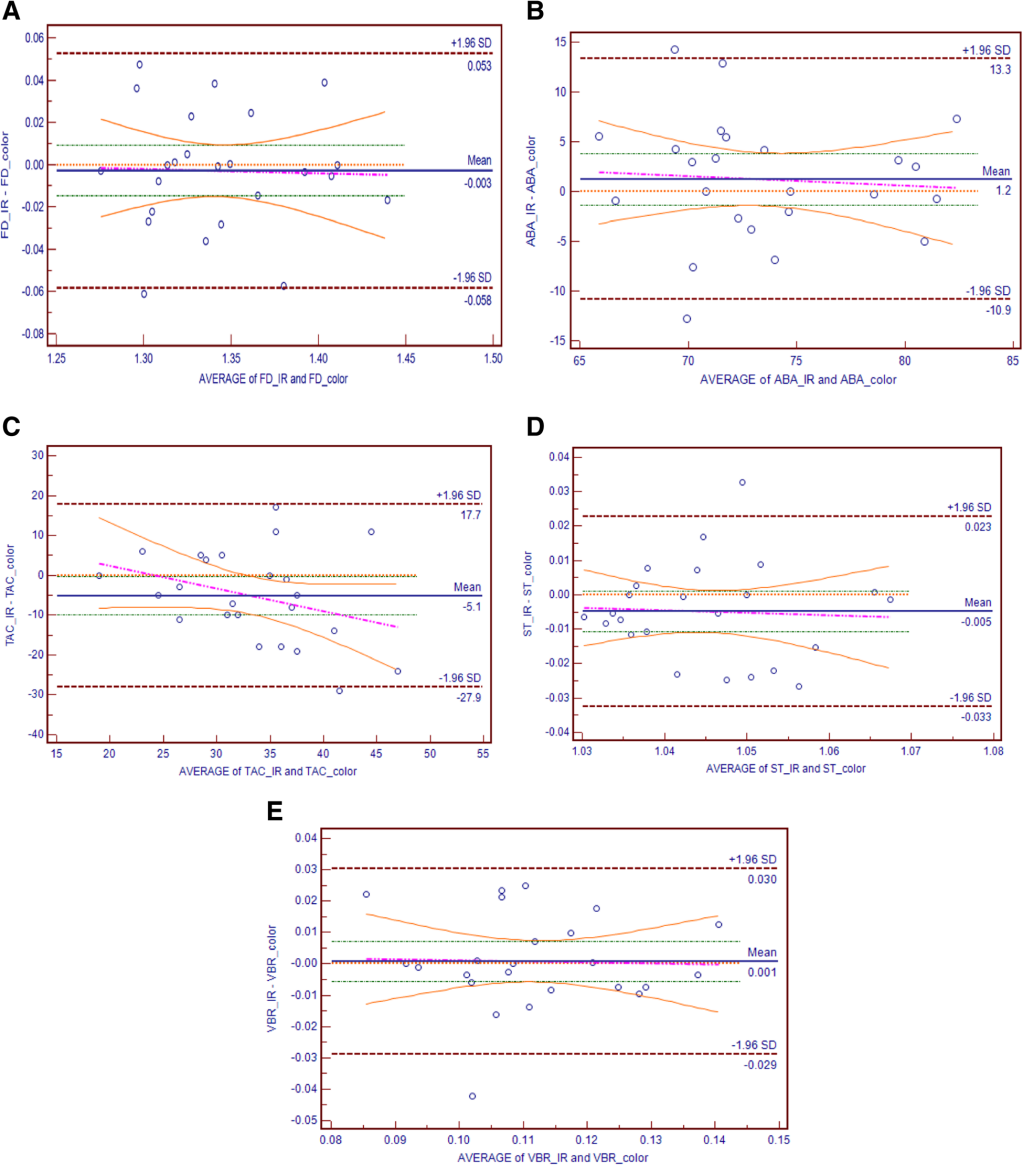
Bland Altman scatter plot of agreement between 1st set of ground truth Color and IR images (*n* = 24): **a** FD; (**b**) ABA; (**c**) TAC; (**d**) ST; (**e**)VBR

The κ statistics between the Color and IR for the first set of ground truth based on RIVAS vessel features were: FD = 0.59, VBR = 0.42, ABA = 0.28, TAC = 0.05 and ST = 0.20. For the second set of ground truth the κ value for the vessel features were: FD = 0.25, VBR = 0.29, ABA = 0.22, TAC = 0.04 and ST = 0.25. The average κ value observed for the first and second ground truth was 0.30 and 0.21 respectively indicating a fair agreement between color and IR images based on RIVAS vessel features. It was found that five retinal vessel features i.e. FD, ABA, TAC, ST and VBR obtained from IR images agreed with color image and could be used interchangeably. Figure 4 shows the BA plot along with the regression line for the five vessel features extracted from RIVAS using manually segmented Color and IR images.

The results for the agreement test between the vessel calibre measurements for the two modalities are shown in Table 2. The mean difference between the Color and the IR was 8.6 (95% LOA 87.2 to − 70.0) for CRAE measurement, and 44.7 (95% LOA 242.1 to − 152.8) for CRVE measurement. The paired sample t-test between Color and IR for CRAE and CRVE showed (*p* values > 0.05) indicating the absence of fixed bias. The regression values, *r*, for the vessel calibre measurement (CRAE = 0.32, 95% LOA -0.42 to 1.08 and CRVE = − 0.38, 95% LOA -0.60 to 1.38) indicates a positive correlation with no presence of proportional bias.

**Table 2:** Agreement analysis between Color and IR images based on summary retinal vessel calibre

Parameter	Mean Difference	LOA	*p* value *	*p* value**	κ ***
CRAE	8.6	87.2 to −70.0	0.29	0.37	0.10
CRVE	44.7	242.1 to −152.8	0.05	0.42	−0.01

*p value of one sample t-tests (comparing between mean difference and zero value) to indicate presence of systemic bias

**p value of Regression Analysis to indicate presence of proportional bias

***κ Cohen’s kappa value to indicate the level of agreement color and IR images

The *p*-value for the *t*-test and the Linear Regression shows that there is no significant difference in terms of vessel calibre between the Colour and IR images.

The κ statistics between the Color and IR for vessel caliber features were: CRAE = 0.10 and CRVE = − 0.01 respectively thus indicating a low significance level.

**Fig. 5:**
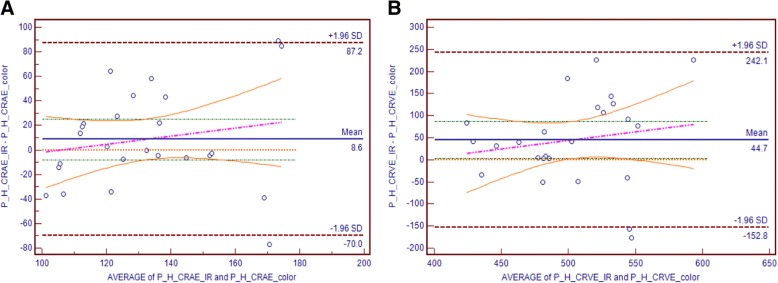
Bland Altman plot of agreement between Color and IR images in terms of Vessel Calibre (*n* = 24): **a** CRAE; (**b**) CRVE

Figure 5. shows the BA plot along with the regression line for the vessel calibre features i.e. CRAE and CRVE obtained from IVAN using Color and IR images.

## Discussion

Retinal vasculature is routinely investigated using the color fundus images for detection of superficial retinal diseases and alterations to the retinal vessels associated with systemic diseases such as diabetes, stroke and cardiovascular diseases. For diseases affecting the deeper layer which requires the examination of cross section of the retina i.e. age-related macular degeneration and glaucoma, OCT is used. It also has in built IR-SLO which has been shown to be superior to the color fundus imaging for detection of diseases such as Choroidal Neovascularization CNV [32]. The system provides the simultaneous information about the tomographic and topological alterations in the retina tissue. While the tomographic information obtained from OCT is routinely used, the IR image that is produced alongside has not been investigated for diagnostic purposes.

Significance of IR imaging is that it can penetrate deeper through the retinal tissue making it suitable for early detection of diseases such as CNV, Cystoid Macular edema and age-related macular degeneration [33, 34]. These images have similar information as the eye-fundus images and the use of these could potentially save time because these are obtained alongside with the OCT. However, they have never been investigated to determine an agreement with the fundus images.

This study has tested and confirmed the agreement between IR and colour eye-fundus images for the 7 retinal vasculature parameters: FD (Box counting), ABA, ST, TAC, VBR, CRAE and CRVE. The study compared color and IR eye-fundus images of 16 volunteers who did not have any ocular disease and statistical tests showed that there was no significant difference between the images. There was also no evidence of statistically significant difference between the two between the 2 groups of data. This shows that IR images obtained using the IR-SLO can be used instead of color eye-fundus images for investigating the retinal vasculature.

This work shows that it is possible to use the IR-SLO feature of OCT to investigate the retinal vasculature. It is thus possible to use IR images of the retina to measure the retinal vasculature features. This may be suitable for people with opacity due to cataract or with people hyper-sensitive to flash of light.

One limitation of this study is the sample size which was small and can result in the misinterpretation of the results because the confidence interval (CI) and limits of agreement (LOA) appear to be higher. Another limitation of this study is that the regression line for few vessel parameters i.e. ABA, TAC and vessel caliber was not horizontal and this indicates the presence of bias effecting the power of agreement between the Color fundus and IR images. The Cohen’s kappa (κ*)* for few vessels features i.e., CRAE and CRVE was found low. All these factors have influenced the strength of agreement resulting in a fair agreement between two imaging system color fundus and IR based on retinal vessel features. Future studies with appropriate number of participants (~ 90) is needed to test the agreement between color and IR retina images.

The outcome of this work has the potential for imaging people with opacity or hypersensitivity to the flash of light, but no such individuals have been tested. This is a limitation of this study, and it is recommended that future studies should investigate people with these conditions.

## Conclusion

It has been observed that there is a fair agreement between the Color and IR fundus images based on the retinal vessel features. The significant outcome of this research is that it validates the application of IR fundus images recorded with the OCT using the IR-SLO for investigating the superficial retinal vasculature and obtain better understanding of pathogenesis. This work indicates that a patient undergoing OCT does not have to be further investigated using color fundus photography, thereby saving the patient the discomfort and health care system the extra expense. Another advantage of this technique IR-SLO does not require optical flash which is required for the color eye-fundus imaging. However, while the major potential application of this work is for investigating the retinal vasculature of people with cataract or similar opacity, these patients have yet not been investigated which is a limitation and would be examined in the future.

## Abbreviations

CRAE: Central Retinal Arteriolar Equivalent
CRVE: Central Retinal Venular Equivalent
FD: Fractal Dimension
IR-SLO: Infrared Scanning Laser Ophthalmoscope
OCT: Optical Coherence Tomography
ST: Simple Tortuosity
TAC: Total Angle Count
VBR: Vessel to background ratio
